# Peroxisome Proliferator-Activated Receptors Alpha, Beta, and Gamma mRNA and Protein Expression in Human Fetal Tissues

**DOI:** 10.1155/2010/690907

**Published:** 2010-07-26

**Authors:** Barbara D. Abbott, Carmen R. Wood, Andrew M. Watkins, Kaberi P. Das, Christopher S. Lau

**Affiliations:** Toxicity Assessment Division, Developmental Toxicology Branch, National Health and Environmental Effects Research Laboratory, (MD-67), Office of Research and Development, US Environmental Protection Agency, Research Triangle Park, NC 27711, USA

## Abstract

Peroxisome proliferator-activated receptors (PPARs) regulate lipid and glucose homeostasis, are targets of pharmaceuticals, and are also activated by environmental contaminants. Almost nothing is known about expression of PPARs during human fetal development. This study examines expression of PPAR*α*, *β*, and *γ* mRNA and protein in human fetal tissues. With increasing fetal age, mRNA expression of PPAR*α* and *β* increased in liver, but PPAR*β* decreased in heart and intestine, and PPAR*γ* decreased in adrenal. Adult and fetal mean expression of PPAR*α*, *β*, and *γ* mRNA did not differ in intestine, but expression was lower in fetal stomach and heart. PPAR*α* and *β* mRNA in kidney and spleen, and PPAR*γ* mRNA in lung and adrenal were lower in fetal versus adult. PPAR*γ* in liver and PPAR*β* mRNA in thymus were higher in fetal versus adult. PPAR*α* protein increased with fetal age in intestine and decreased in lung, kidney, and adrenal. PPAR*β* protein in adrenal and PPAR*γ* in kidney decreased with fetal age. This study provides new information on expression of PPAR subtypes during human development and will be important in evaluating the potential for the developing human to respond to PPAR environmental or pharmaceutical agonists.

## 1. Introduction

Peroxisome proliferator-activated receptors (PPARs) belong to the nuclear hormone receptor superfamily and there are three primary subtypes (*α*, *β*/*δ*, and *γ*) [1]. These receptors play important roles in embryonic and fetal development as well as placental function [2, 3], regulating many cellular and metabolic processes [4]. PPARs control energy homeostasis, are important regulators of adipogenesis, lipid metabolism, inflammatory responses, and hematopoiesis, and are implicated in chronic diseases such as diabetes, obesity and atherosclerosis [5–8]. PPAR*β* and *γ* have roles in early embryonic survival and implantation [9, 10]. PPARs regulate gene expression by binding to specific DNA sequences, peroxisome proliferator response elements (PPREs), in the promoter regions of target genes. Prior to DNA binding PPAR forms a heterodimer with the retinoid X receptor (RXR) [11, 12]. A number of endogenous ligands have been identified for each PPAR subtype, including long-chain fatty acids, polyunsaturated fatty acids such as linoleic and arachidonic acids, saturated fatty acids, and eicosanoids [1]. A variety of synthetic ligands have been developed for pharmaceutical purposes to treat chronic diseases such as hyperlipidemia, diabetes, and metabolic syndrome. In addition, some chemicals and environmental contaminants activate PPARs, for example, phthalates, tri- and dichloroacetic acids, trichloroethylene, and the perfluorinated alkyl and sulfonyl acid compounds (PFAAs) [11–14]. 

PFAAs, including perfluorooctanoic acid (PFOA), perfluorononanoic acid (PFNA), and perfluorooctane sulfonate (PFOS), are highly stable molecules with chemical properties that make them excellent surfactants [15]. For many years these chemicals were widely used in industrial applications and are now found as persistent environmental contaminants that are also present in the tissues and serum of wildlife and humans [15–17]. In laboratory studies, prenatal exposure of rodents to these compounds produces dose-related effects on pre- and postnatal survival, developmental delay, and deficits in postnatal growth [18–23]. PFAAs activate PPAR*α*, and the developmental toxicity of PFOA and PFNA in the mouse was shown to be dependent on expression of PPAR*α* in the fetus (effects of prenatal exposure to PFOA or PFNA that occur in wild-type mice were not observed in PPAR*α* knockout offspring) [24, 25]. PPARs are expressed in the mouse embryo and fetus (reviewed in [26]), and prenatal exposure to PFAAs was shown to change gene expression in the pre- and postnatal livers in a pattern indicative of activation of PPAR as well as the CAR nuclear receptor [27–29]. 

Almost nothing is known about expression of PPAR during human development. At the present time, a search of the literature revealed only one paper that described the expression of PPARs in the human fetus, and that paper described expression in the gastrointestinal (GI) tract [30]. This gap in scientific knowledge of PPAR expression during human development requires attention as PFAAs, which activate PPAR, alter gene expression, and have developmental toxicity in the rodent, are pervasive in the environment and have been found in serum and blood samples of populations around the world, including samples from infants, children, and in umbilical cord blood and milk (indicating prenatal and postnatal exposure of infants) [17, 31–36]. Thus, in order to evaluate the potential for these environmental compounds, and others, to affect human fetal development, it is important to have information regarding the expression of PPARs in the developing human fetus. The present study reports mRNA and protein expression for PPAR*α*, *β*, and *γ* in embryonic day (ED) 54 to 125 human fetal liver, heart, lung, kidney, stomach, intestine, adrenal, spleen, and thymus.

## 2. Methods and Materials

### 2.1. Human Fetal and Adult Samples

 Human fetal tissues ranging in age from embryonic day (ED) 54 to 125 were obtained from the Birth Defects Research Laboratory at the University of Washington, Seattle. The collection of tissue specimens from clinically aborted fetuses by the Birth Defects Research Laboratory (including informed consent for the donation and all procedures) was conducted with Human Subjects Institutional Review Board (IRB) approval. At the EPA, the study was reviewed by the Office of Human Research Ethics, UNC Biomedical IRB, and approved by the National Health Effects and Environmental Research Laboratory (NHEERL) Human Research Protocol Office (HRPO).

Tissues were snap frozen as soon as possible after collection and stored at −80°C until shipped on dry ice. On arrival at EPA, samples were stored at −80°C until processed for total RNA and protein. The nine tissues analyzed included liver, heart, lung, kidney, stomach, intestine, adrenal, spleen, and thymus. Prior to processing the fetal tissues to prepare RNA and protein, samples were weighed and smaller samples were designated for RNA preparation only, while larger samples were divided for both RNA and protein preparation, and any excess sample was returned (still frozen) for storage at −80°C. Handling during the weighing and division of samples was done over dry ice to the extent possible to minimize thawing. Adult total RNA for the 9 tissues examined in the study was obtained from FirstChoice Human Total RNA Survey Panel, Ambion, Inc, (each adult sample consisted of pooled total RNA from 3 individuals). In addition, tissue samples from 23 adult human livers were available for comparison of PPAR mRNA expression in adult and fetal liver. These samples were obtained from CellzDirect, Inc. (Durham, NC). Total RNA was prepared from the frozen adult liver tissue samples and qPCR performed, as described for the fetal samples.

### 2.2. qPCR Experimental Design and Procedures

 Each tissue was run in separate qPCR experiments (e.g., liver samples were not run with those of any other tissue). In the qPCR experiments, expression of PPAR*α*, *β*, *γ* and an internal control gene were examined on each plate, and samples on the plate included 2 replicates of each fetal sample and of the appropriate pooled adult tissue (FirstChoice Human Total RNA). In cases where there were too many samples of a tissue to run all of the reactions on one plate, the samples were run across 2 plates such that each age was represented as equally as possible on each plate. The actual number of samples examined for each tissue is stated in the results section, but the number available ranged from 23 to 46 specimens, except for thymus which had 11 specimens. PPAR gene expression was expressed relative to an internal control gene. The fetal samples of each tissue were examined for expression of *β*-actin, *β*2-microglobulin, and glyceraldehyde-3-phosphate dehydrogenase (GAPDH), as potential internal control genes. Regression analysis of cycle threshold (Ct) was performed for each potential control gene to detect any changes in expression with age. Based on favorable regression outcomes in all of the tissues (no significant change with age), *β*2-microglobulin (B2M) was selected as the internal control gene (data not shown). 

Tissue was homogenized and extracted in TRI Reagent (Sigma Chemical, St. Louis, MO) according to the manufacturer's directions, and RNA pellets were stored in 70% ethanol at −80°C until further use. Following resuspension in nuclease-free water (Promega Corporation, Madison, WI), the RNA was quantified and evaluated for purity (260 nm/280 nm and 260 nm/230 nm ratio) using a NanoDrop ND-1000 spectrophotometer (NanoDrop Technologies,Wilmington, DE). Prior to qPCR, 2 *μ*g total RNA was digested using 2 units of DNaseI (Promega Corporation, Madison, WI) for 30 min at 37°C followed by 10 min at 65°C in a buffer containing 40 mM Tris (pH 8.0), 10 mM MgSO_4_, and 1 mM CaCl_2_. The DNase-treated RNA was then quantified using a Quant-iT RiboGreen RNA assay kit according to the manufacturer's protocol (Invitrogen Corporation, Carlsbad, CA). Approximately 1 *μ*g of the DNase-treated RNA was reverse transcribed using a High-Capacity cDNA Archive Kit according to the provided protocol (Applied Biosystems). Amplification was performed on an Applied Biosystems model 7900HT Fast Real-Time PCR System in duplicate using 25 ng cDNA and TaqMan Universal PCR Master Mix (Applied Biosystems) in a total volume of 12 *μ*l. The following TaqMan assays (Applied Biosystems) were included in the study: PPAR*α* (Hs00947539_m1), PPAR*β* (Hs00602622_m1), PPAR*γ* (Hs00234592_m1), *β*-actin (Hs99999903_m1), GAPDH (Hs99999905_m1), *β*-2 microglobulin (Hs99999907_m1). 

PPAR mRNA Ct values, calculated by Applied Biosystems SDS2.2.2 software, were normalized by subtraction of the Ct for the internal control, B2M, generating *δ*Ct values. The mean *δ*Ct for each sample was calculated from the 2 replicates and then analyzed to evaluate changes in expression with fetal age (regression analysis). Differences in expression between subtypes were determined using ANOVA of all mean *δ*Ct values (without regard to age), with Bonferroni's post-test applied for pairwise comparisons (Prism 4.0, GraphPad Software, San Diego, CA). Data are graphed as a log plot of 2^−*δ*Ct^. For all tissues except liver, a comparison of the fetal samples with the single adult pooled sample was performed using Ct values and a *t*-distribution test (sample size less than 30) or the *Z*-distribution test (sample size equal to or greater than 30) to determine the probability that an adult value of this extreme or more extreme would be found in the distribution of fetal values (using probability calculators available on-line at http://faculty.vassar.edu/lowry/tabs.html or http://davidmlane.com/hyperstat/z_table.html). As the internal control gene expression was not the same in adult and fetal tissues, Ct values, and not *δ*Ct values, were used for this comparison. Data comparing fetal (mean Ct of 2 replicates) and adult Ct (each replicate shown in the plot) are graphed as a vertical scatter plot of Ct. For the liver, there were 23 adult liver specimens available for comparison with the fetal liver samples. The adult samples were from both males and females and ranged in age from 21 to 86 years, but analysis of either Ct or dCt showed no effect of either sex or age on the expression of PPAR*α*, *β*, or *γ* or B2M (data not shown). The adult and fetal liver samples had comparable levels of the internal control gene, B2M, and thus it was possible to compare the normalized Ct values (dCt) using ANOVA with Bonferroni's post-test applied for pairwise comparisons (Prism 4.0, GraphPad Software, San Diego, CA).

### 2.3. Western Blot Experimental Design and Procedures

 Samples of each tissue were run in separate Western blot experiments (e.g., liver samples were not run with those of any other tissue). In general, most of the tissues required 2-3 Western blots to accommodate all of the samples (only 12 sample lanes were available per gel), and the samples were blocked across blots such that the age range was represented as uniformly as possible on each blot. The actual number of samples examined for each tissue is stated in the results section, but the number available ranged from 5 to 36 specimens, and only thymus and spleen had fewer than 22 specimens. Each blot was examined for expression of one of the PPAR subtypes and for the internal control protein. GAPDH was selected as the internal control protein as expression did not change with age (based on regression analyses of GAPDH protein expression across age in each of the 9 tissues, data not shown). A positive control for antibody detection of PPAR*α*, *β*, or *γ* was also run on each blot. Positive controls were Hep G2 whole cell extract (Santa Cruz, SC-2227), Jurkat cell nuclear extract (Santa Cruz, SC-2132), and U937 whole cell extract (Santa Cruz, SC-2239), for expression of PPAR*α*, *β*, or *γ*, respectively. After all tissues were examined in this manner, additional Western blots were run in which all 9 tissues were represented on the blot, with most of the tissues from the same 91-day-old fetus (to the extent possible, as not all tissues were available from any single fetus, and thymus was not available from a 91-day fetus). Three “across-tissue” blots were run for each PPAR subtype (i.e., *n* = 3 fetuses per tissue examined in the “across-tissue” survey).

Western blots were run with 25 *μ*g of total cell lysate or positive control per lane. All gels were 7.5% acrylamide precast gels (Biorad, Hercules, CA) and were run for 90 min at 125 V. Protein transfer to nitrocellulose membrane (Biorad) was done for 40 min using the Biorad semidry transfer system. Membranes were blocked for 1 hr in 5% milk and incubated overnight in primary antibody in 5% milk. Primary antibodies for PPAR*α* (SC-9000) and PPAR*γ* (SC-7196) were obtained from Santa Cruz Biotechnologies (Santa Cruz, CA) and used at a dilution of 1:200. PPAR*β* (Abcam 21209) antibody was obtained from Abcam, Inc, (Cambridge, MA) and used at 1 : 750 dilution. Antibody for GAPDH (SC-25778) was from Santa Cruz Biotechnologies and was diluted at 1 : 10,000. After overnight incubation with primary antibody, blots were probed 1 hr with a horseradish peroxidase-conjugated secondary antibody in 5% milk. Secondary antibodies were diluted 1 : 5000 and included goat anti-rabbit Jax 111-035-144 (West Grove, PA), goat anti-rabbit KPL 074-1506 and rabbit anti-goat KPL 14-13-06 (KPL, Gaithersburg, MD). Chemiluminescence was imaged using a Biorad Fluor-S machine with 2 or 5 min exposures. Biorad Quantity One software was used to perform volume rectification densitometry with background subtraction on the chemiluminescence images, generating data for both the PPAR and GAPDH bands. PPAR protein expression was expressed relative to the internal control gene, GAPDH, and regression analysis of the relative values was performed to detect any significant change in slope with age (Prism 4.0, GraphPad Software, San Diego, CA).

## 3. Results

The expression of PPARs is presented for each tissue, reporting any change in expression of protein or mRNA with gestational age, comparing the relative level of mRNA expression of the isotypes, and comparing mRNA expression in the fetal organ to that observed in the human adult sample of that tissue. Protein and mRNA for all PPAR isotypes were detected in all of the 9 tissues and the results are summarized in Table 1, which also lists the tissues from highest to lowest expression of mRNA for each PPAR. Table 2 summarizes the relative expression of the isotypes within each tissue. The data is shown using the same presentation format for each tissue. A full narrative presentation is given for the first tissue presented (liver) and, for the sake of brevity, the results for other tissues omit repetitive explanations which apply to all the data sets. 

### 3.1. Liver

**Figure 1:**
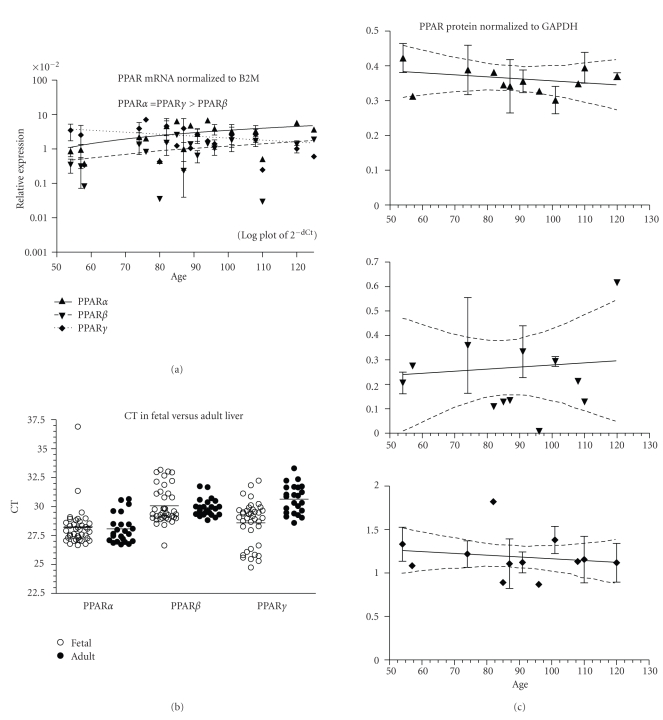
Liver. (a) The expression of PPAR*α*, *β*, and *γ* mRNA is shown across the fetal age range. Log plot of mean ± SEM Ct is normalized to *β*-2-microglobulin (B2M). (b) The fetal expression of PPAR*α*, *β*, and *γ* is shown relative to expression in adult liver. Each symbol represents the mean Ct value of 2 replicates for each fetal (open circles) and adult (filled circles) sample (overall mean for each group is shown as a horizontal line). (c) PPAR*α*, *β*, and *γ* protein expression is shown across the fetal age range. Western blot density is normalized to glyceraldehyde-3-phophate dehydrogenase (GAPDH). Up arrowhead indicates PPAR*α*, down arrowhead PPAR*β*, and diamond PPAR*γ*. If only one sample was available for a particular age, then an error term could not be calculated and no SEM bar is shown. Regression analysis evaluated change with age. Dashed lines in graphs of C are the 95% confidence interval.

 Human fetal liver expressed PPAR*α*, *β*, and *γ* mRNA from ED54–125 (data acquired from 39 fetuses). Expression of PPAR*α* and PPAR*β* increased significantly while PPAR*γ* remained unchanged across the age range (Figure 1(a), *P* < .01 and .05, resp.).Expression levels of PPAR*α* and *γ* across all ages were not significantly different and both were more highly expressed than PPAR*β* (*P* < .001). *β*2M mRNA expression was considerably higher than any of the PPAR subtypes (mean ± SEM Ct across all ages, note that a lower Ct signifies more abundant mRNA than a high Ct: *β*2M = 22.4 ± 0.3, PPAR*α* = 28.2 ± 0.3, PPAR*β* = 30.1 ± 0.3, PPAR*γ* = 28.6 ± 0.3). The expression of PPAR mRNA in the fetal liver was compared to that in 23 adult human liver samples. The adult samples were from both males and females and ranged in age from 21 to 86 years, but analysis showed no effect of either sex or age on the expression of PPAR*α*, *β*, *γ*, or B2M (data not shown). The expression of B2M, the internal control gene, in adult and fetal liver samples was not significantly different (21.9 ± 0.2, 22.4 ± 0.3, mean ± SEM, resp.), and thus it was possible to analyze PPAR values normalized to B2M (dCt). Analysis of unadjusted Ct or dCt gave the same outcomes, and Figure 1(b) shows plots of the Ct values for adult and fetal livers. The human fetal and adult livers expressed PPAR*α* and *β* mRNA at levels that were not significantly different (Figure 1(b)), but PPAR*γ* was significantly higher in the fetal liver (*P* < .001, lower mean Ct indicates higher mRNA present in a sample). The overall outcome for PPAR*α* and *β* was the same from the pooled total RNA from 3 donors (Ambion FirstChoice liver sample, data not shown) as that from the 23 individuals; adult and fetal liver expression did not significantly differ. The Ambion FirstChoice pooled adult liver RNA indicated that PPAR*γ* did not differ between adult and fetal livers, but the data from the 23 individuals showed a significantly higher expression in the fetus, and the larger “*n*” of that assay would lend support to the validity of that outcome. In the fetal liver, PPAR*α*, *β*, and *γ* protein expression did not change with fetal age (Figure 1(c); data from 22 fetuses, ED54–120).

### 3.2. Heart

**Figure 2:**
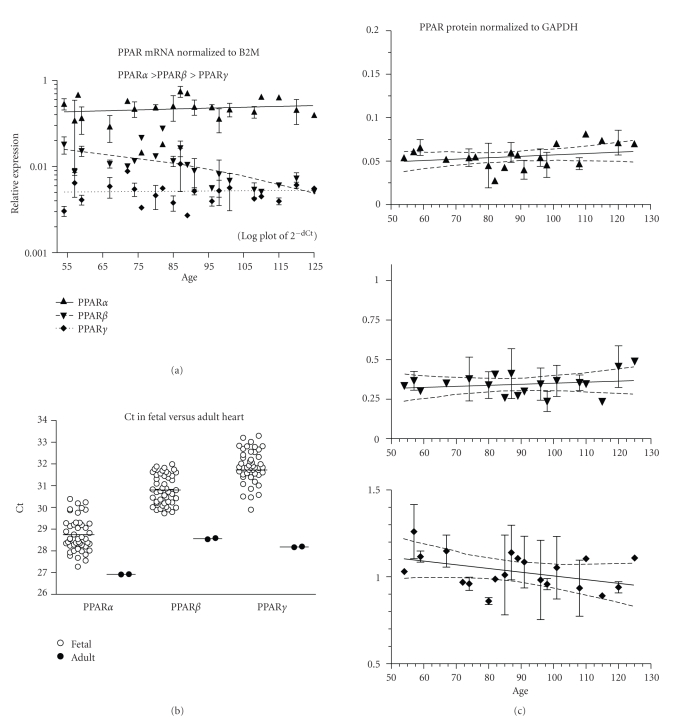
Heart. (a) The expression of PPAR*α*, *β*, and *γ* mRNA is shown across the fetal age range. Log plot of mean ± SEM Ct is normalized to *β*-2-microglobulin (B2M). (b) The fetal expression of PPAR*α*, *β*, and *γ* is shown relative to expression in adult heart. Each symbol represents the mean Ct value of 2 replicates for each fetal (open circles) sample and adult (filled circles) individual replicates are shown (overall mean for each group is shown as a horizontal line). (c) PPAR*α*, *β*, and *γ* protein expression is shown across the fetal age range. Western blot density normalized to glyceraldehyde-3-phophate dehydrogenase (GAPDH). Up arrowhead indicates PPAR*α*, down arrowhead PPAR*β*, and diamond PPAR*γ*. If only one sample was available for a particular age, then an error term could not be calculated and no SEM bar is shown. Regression analysis evaluated change with age. Dashed lines in graphs of C are the 95% confidence interval.

 PPAR*α* and *γ* expression did not change, but PPAR*β* expression decreased (*P* < .0001) with fetal age (Figure 2(a), ED54–125, *n* = 46 fetuses).PPAR*α*, PPAR*β*, and PPAR*γ* relative expressions are shown in Table 2 and differences between isotypes were significant at *P* < .001. *β*2M mRNA expression was higher than PPAR (mean Ct ± SEM: *β*2M = 24.2 ± 0.8, PPAR*α* = 28.7 ± 0.1, PPAR*β* = 30.8 ± 0.1, and PPAR*γ* = 31.9 ± 0.1). Fetal PPAR*α*, *β*, and *γ* mRNA expression was lower than that of the adult sample (Figure 2(b); *P* < .05,.001,.001, resp.). PPAR protein expression did not change with fetal age (Figure 2(c); 36 fetuses, ED54–125).

### 3.3. Lung

**Figure 3:**
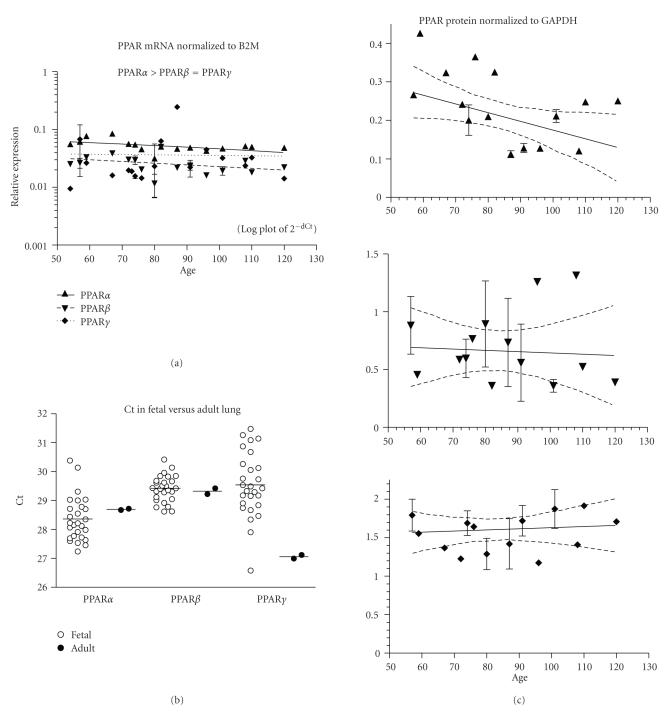
Lung. (a) The expression of PPAR*α*, *β*, and *γ* mRNA is shown across the fetal age range. Log plot of mean ± SEM Ct is normalized to *β*-2-microglobulin (B2M). (b) The fetal expression of PPAR*α*, *β*, and *γ* is shown relative to expression in adult lung. Each symbol represents the mean Ct value of 2 replicates for each fetal (open circles) sample and adult (filled circles) individual replicates are shown (overall mean for each group is shown as a horizontal line). (c) PPAR*α*, *β*, and *γ* protein expression is shown across the fetal age range. Western blot density normalized to glyceraldehyde-3-phophate dehydrogenase (GAPDH). Up arrowhead indicates PPAR*α*, down arrowhead PPAR*β*, and diamond PPAR*γ*. If only one sample was available for a particular age, then an error term could not be calculated and no SEM bar is shown. Regression analysis evaluated change with age. Dashed lines in graphs of C are the 95% confidence interval.

 PPAR mRNA expression in fetal lung did not change with age (Figure 3(a), ED54–120, *n* = 27 fetuses). PPAR*α* was the most highly expressed isotype (*P* < .001, Table 2). *β*2M mRNA expression was higher than PPAR, (mean Ct ± SEM: *β*2M = 24.0 ± 0.2, PPAR*α* = 28.4 ± 0.1, PPAR*β* = 29.4 ± 0.1, and PPAR*γ* = 29.5 ± 0.2). Fetal and adult PPAR*α* and *β* mRNA expressions were not different, but fetal PPAR*γ* was lower (Figure 3(b); *P* < .05). PPAR*α* protein levels decreased (*P* < .05), but PPAR*β* and *γ* did not change with fetal age (Figure 3(c), ED57 to 120, *n* = 27 fetuses).

### 3.4. Kidney

**Figure 4:**
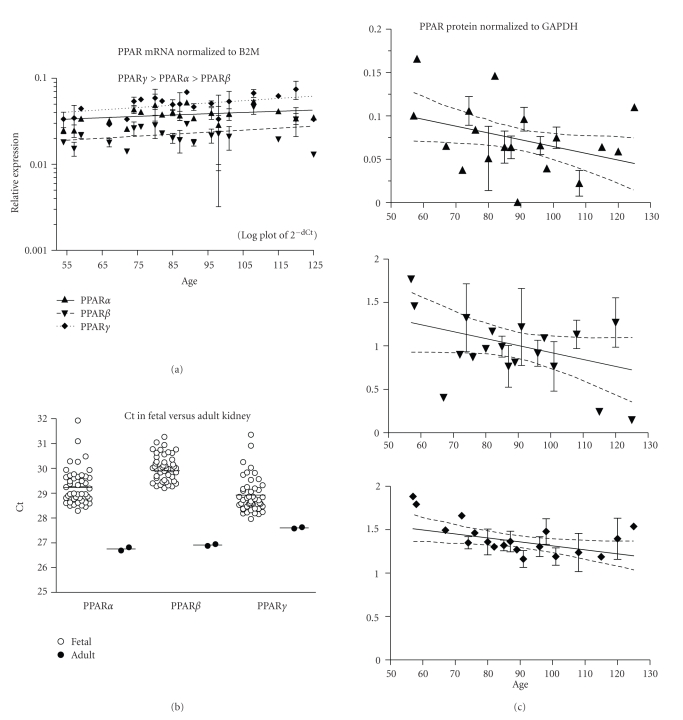
Kidney. (a) The expression of PPAR*α*, *β*, and *γ* mRNA is shown across the fetal age range. Log plot of mean ± SEM Ct is normalized to *β*-2-microglobulin (B2M). (b) The fetal expression of PPAR*α*, *β*, and *γ* is shown relative to expression in adult kidney. Each symbol represents the mean Ct value of 2 replicates for each fetal (open circles) sample and adult (filled circles) individual replicates are shown (overall mean for each group is shown as a horizontal line). (c) PPAR*α*, *β*, and *γ* protein expression is shown across the fetal age range. Western blot density normalized to glyceraldehyde-3-phophate dehydrogenase (GAPDH). Up arrowhead indicates PPAR*α*, down arrowhead PPAR*β*, and diamond PPAR*γ*. If only one sample was available for a particular age, then an error term could not be calculated and no SEM bar is shown. Regression analysis evaluated change with age. Dashed lines in graphs of C are the 95% confidence interval.

 PPAR mRNA expression did not change with age (Figure 4(a), 46 fetuses, ED54–125). PPAR*γ* expression was higher than PPAR*α* and PPAR*β* (*P* < .05, *P* < .001, resp.), and PPAR*α* was higher than PPAR*β* (*P* < .001). *β*2M mRNA expression was higher than PPAR (mean Ct ± SEM: *β*2M = 24.5 ± 0.1, PPAR*α* = 29.3 ± 0.1, PPAR*β* = 30.0 ± 0.1, PPAR*γ* = 28.9 ± 0.1). PPAR*α* and *β* fetal mRNAs were lower than in the adult (Figure 4(b); *P* < .01, <.0001, respectively), but PPAR*γ* was similar (*P* = .07). PPAR*β* protein expression did not change with fetal age (*P* = .09), but PPAR*α* and *γ* decreased (*P* < .05, Figure 4(c); 36 fetuses, ED57–125).

### 3.5. Stomach

**Figure 5:**
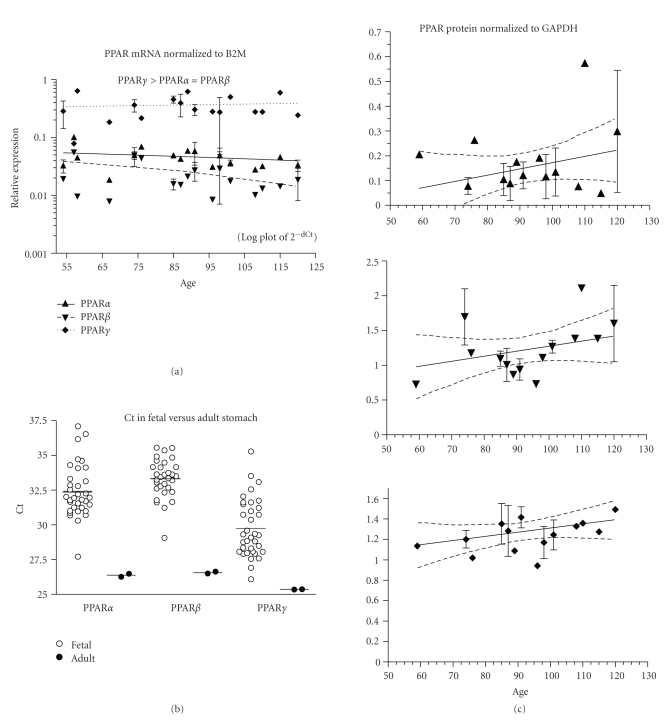
Stomach. (a) The expression of PPAR*α*, *β*, and *γ* mRNA is shown across the fetal age range. Log plot of mean ± SEM Ct is normalized to *β*-2-microglobulin (B2M). (b) The fetal expression of PPAR*α*, *β*, and *γ* is shown relative to expression in adult stomach. Each symbol represents the mean Ct value of 2 replicates for each fetal (open circles) sample and adult (filled circles) individual replicates are shown (overall mean for each group is shown as a horizontal line). (c) PPAR*α*, *β*, and *γ* protein expression is shown across the fetal age range. Western blot density normalized to glyceraldehyde-3-phophate dehydrogenase (GAPDH). Up arrowhead indicates PPAR*α*, down arrowhead PPAR*β*, and diamond PPAR*γ*. If only one sample was available for a particular age, then an error term could not be calculated and no SEM bar is shown. Regression analysis evaluated change with age. Dashed lines in graphs of C are the 95% confidence interval.

 PPAR mRNA expression did not change with age (Figure 5(a), 35 fetuses, ED54–120). PPAR*γ* was the most highly expressed isotype (*P* < .001, Table 2). *β*2M mRNA expression was higher than PPAR (mean Ct ± SEM: *β*2M = 28.0 ± 0.3, PPAR*α* = 32.4 ± 0.3, PPAR*β* = 33.3 ± 0.3, and PPAR*γ* = 29.8 ± 0.4). PPAR*α*, *β*, and *γ* mRNA expression was lower in fetal than in adult stomach (Figure 5(b); *P* < .01, <.0001, <.05, resp.). PPAR protein expression did not change with fetal age (Figure 5(c); 26 fetuses, ED59–120).

### 3.6. Intestine

**Figure 6:**
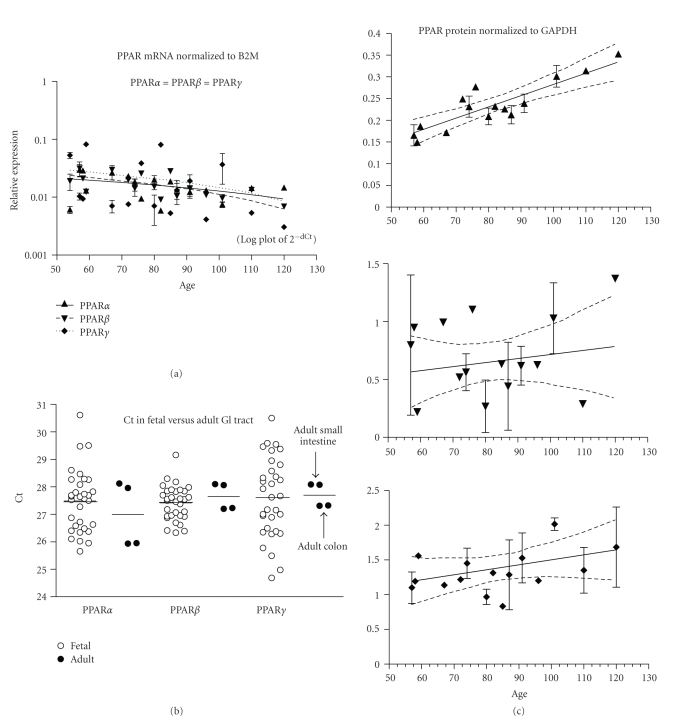
Intestine. (a) The expression of PPAR*α*, *β*, and *γ* mRNA is shown across the fetal age range. Log plot of mean ± SEM Ct is normalized to *β*-2-microglobulin (B2M). (b) The fetal expression of PPAR*α*, *β*, and *γ* is shown relative to expression in adult small intestine and colon (filled circles show the 2 replicates of small intestine above and 2 replicates of colon below the line indicating the mean of the combined tissue values). Each open symbol represents the mean Ct value of 2 replicates for each fetal sample (overall mean is shown as a horizontal line). (c) PPAR*α*, *β*, and *γ* protein expression is shown across the fetal age range. Western blot density is normalized to glyceraldehyde-3-phophate dehydrogenase (GAPDH). Up arrowhead indicates PPAR*α*, down arrowhead PPAR*β*, and diamond PPAR*γ*. If only one sample was available for a particular age, then an error term could not be calculated and no SEM bar is shown. Regression analysis evaluated change with age. Dashed lines in graphs of C are the 95% confidence interval.

 PPAR*α* and *γ* mRNA expression did not change with age, but PPAR*β* decreased (*P* < .001, Figure 6(a), 32 fetuses, ED54–120). PPAR*α*, *β*, and *γ* were expressed at similar levels (Table 2). *β*2M mRNA expression was higher than PPAR (mean Ct ± SEM: *β*2M = 21.4 ± 0.2, PPAR*α* = 27.5 ± 0.2, PPAR*β* = 27.4 ± 0.1, and PPAR*γ* = 27.6 ± 0.3). Fetal intestinal PPAR mRNA was not significantly different from either the adult small intestine or the adult colon (Figure 6(b)). PPAR*α* protein expression increased (*P* < .001), while PPAR*β* and *γ* proteins did not change with fetal age (Figure 6(c); 29 fetuses, ED57–120).

### 3.7. Adrenal

**Figure 7:**
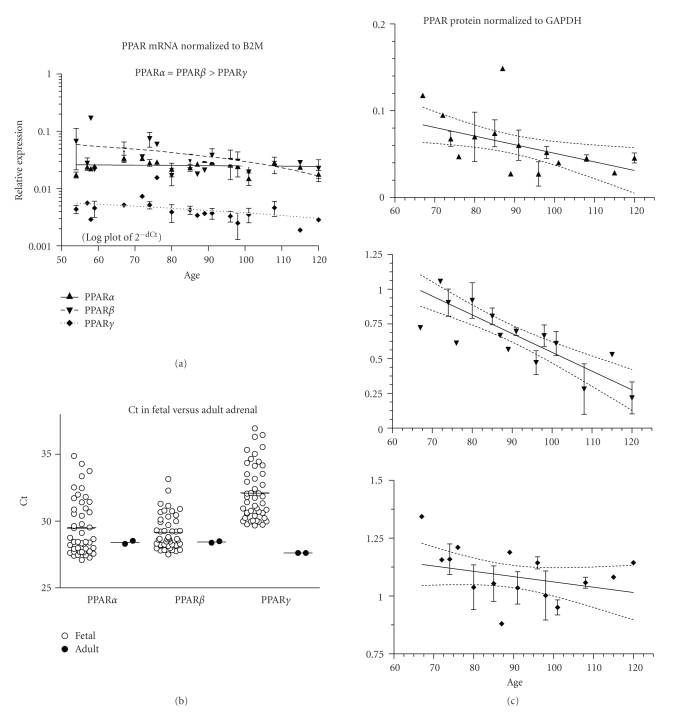
Adrenal. (a) The expression of PPAR*α*, *β*, and *γ* mRNA is shown across the fetal age range. Log plot of mean ± SEM Ct is normalized to *β*-2-microglobulin (B2M). (b) The fetal expression of PPAR*α*, *β*, and *γ* is shown relative to expression in adult adrenal. Each symbol represents the mean Ct value of 2 replicates for each fetal (open circles) sample and adult (filled circles) individual replicates are shown (overall mean for each group is shown as a horizontal line). (c) PPAR*α*, *β*, and *γ* protein expression is shown across the fetal age range. Western blot density is normalized to glyceraldehyde-3-phophate dehydrogenase (GAPDH). Up arrowhead indicates PPAR*α*, down arrowhead PPAR*β*, and diamond PPAR*γ*. If only one sample was available for a particular age, then an error term could not be calculated and no SEM bar is shown. Regression analysis evaluated change with age. Dashed lines in graphs of C are the 95% confidence interval.

 PPAR*γ* mRNA decreased with age (*P* < .05), while PPAR*α* and *β* remained unchanged (although *P* = .0503 for PPAR*β*; Figure 7(a), 46 fetuses, ED54–120). PPAR*α* and *β* mRNAs were more highly expressed than PPAR*γ* (*P* < .001, Table 2). *β*2M mRNA expression was higher than PPAR, (mean Ct ± SEM: *β*2M = 24.2 ± 0.3, PPAR*α* = 29.5 ± 0.3, PPAR*β* = 29.2 ± 0.2, and PPAR*γ* = 32.1 ± 0.3). Fetal and adult PPAR*α* and *β* mRNAs were not different, but PPAR*γ* was lower in fetal adrenal (*P* < .05; Figure 7(b)). PPAR*α* and *β* protein expression decreased with fetal age (*P* < .05, *P* < .001, resp.; Figure 7(c); 36 fetuses, ED67–120).

### 3.8. Spleen

**Figure 8:**
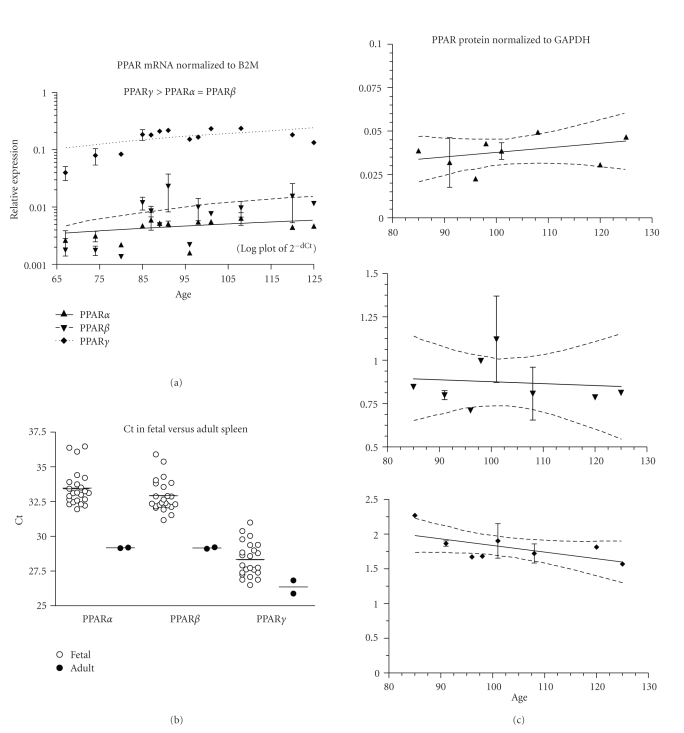
Spleen. (a) The expression of PPAR*α*, *β*, and *γ* mRNA is shown across the fetal age range. Log plot of mean ± SEM Ct is normalized to *β*-2-microglobulin (B2M). (b) The fetal expression of PPAR*α*, *β*, and *γ* is shown relative to expression in adult spleen. Each symbol represents the mean Ct value of 2 replicates for each fetal (open circles) sample and adult (filled circles) individual replicates are shown (overall mean for each group is shown as a horizontal line). (c) PPAR*α*, *β*, and *γ* protein expression is shown across the fetal age range. Western blot density is normalized to glyceraldehyde-3-phophate dehydrogenase (GAPDH). Up arrowhead indicates PPAR*α*, and down arrowhead PPAR*β*, and diamond PPAR*γ*. If only one sample was available for a particular age, then an error term could not be calculated and no SEM bar is shown. Regression analysis evaluated change with age. Dashed lines in graphs of C are the 95% confidence interval.

 PPAR mRNA expression did not change with age (Figure 8(a), 23 fetuses, ED67–125). PPAR*γ* was the most highly expressed isotype (*P* < .001, Table 2). *β*2M mRNA expression was higher than PPAR (mean Ct ± SEM: *β*2M = 25.6 ± 0.2, and PPAR*α* = 33.5 ± 0.3, PPAR*β* = 32.9 ± 0.2, PPAR*γ* = 28.3 ± 0.3). Fetal PPAR*α* and *β* mRNAs were lower than in the adult (*P* < .01, Figure 8(b)). PPAR*α*, *β*, and *γ* protein expression did not change with fetal age (Figure 8(c); 11 fetuses, ED85–125).

### 3.9. Thymus

**Figure 9:**
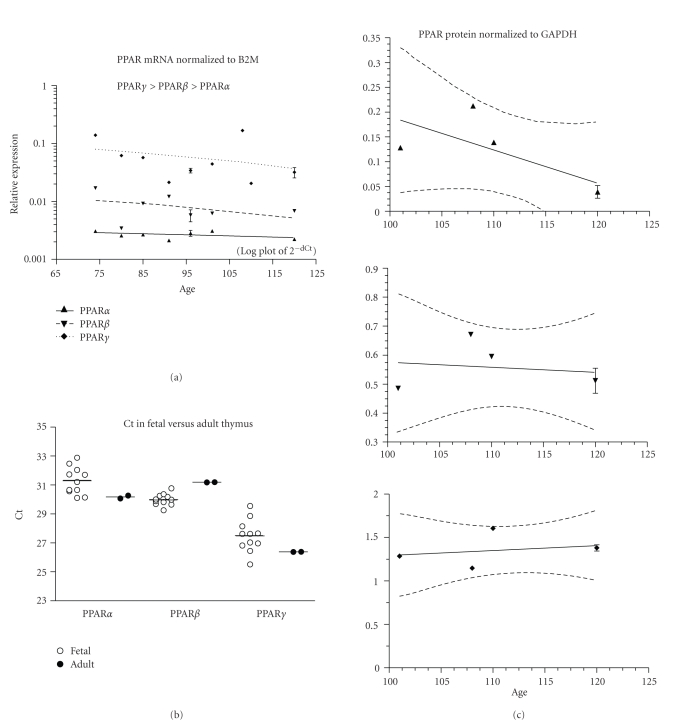
Thymus. (a) The expression of PPAR*α*, *β*, and *γ* mRNA is shown across the fetal age range. Log plot of mean ± SEM Ct is normalized to *β*-2-microglobulin (B2M). (b) The fetal expression of PPAR*α*, *β*, and *γ* is shown relative to expression in adult thymus. Each symbol represents the mean Ct value of 2 replicates for each fetal (open circles) sample and adult (filled circles) individual replicates are shown (overall mean for each group is shown as a horizontal line). (c) PPAR*α*, *β*, and *γ* protein expression is shown across the fetal age range. Western blot density is normalized to glyceraldehyde-3-phophate dehydrogenase (GAPDH). Up arrowhead indicates PPAR*α*, and down arrowhead PPAR*β*, and diamond PPAR*γ*. If only one sample was available for a particular age, then an error term could not be calculated and no SEM bar is shown. Regression analysis evaluated change with age. Dashed lines in graphs of C are the 95% confidence interval.

 PPAR mRNA expression did not change with age (Figure 9(a), 11 fetuses, ED74–120). PPAR*γ* mRNA expression was higher than PPAR*α* or *β* (*P* < .001), and that of PPAR*β* was higher than PPAR*α* (*P* < .01). *β*2M mRNA expression was higher than PPAR (mean Ct ± SEM Ct: *β*2M = 23.1 ± 0.4, PPAR*α* = 31.3 ± 0.3, PPAR*β* = 30.0 ± 0.1, and PPAR*γ* = 27.5 ± 0.3). PPAR*β* fetal mRNA expression was higher than in the adult (*P* < .05, Figure 9(b)). PPAR protein expression did not change with fetal age (Figure 9(c); 5 fetuses, ED101–120).

### 3.10. Comparison of PPAR*α*, *β*, *γ* Expression Levels in Different Tissues

Table 1 lists the tissues in an order based on the level of RNA expression in fetal tissues such that the first tissue listed for each subtype has the highest and the last in the list has the lowest expression. The ranking for RNA expression is based on the mean Ct across all ages for each tissue. Expression of PPAR*α* mRNA is the highest in the intestine, liver, and lung and is relatively low in stomach and spleen. PPAR*β* was the highest in intestine, adrenal, and lung, while expression in spleen and stomach was relatively low. PPAR*γ* was the highest in thymus, intestine, and spleen, but was poorly expressed in heart and adrenal. Among all the tissues, intestine was unique in having high expression of all three subtypes, although lung expressed high levels of both PPAR*α* and *β*. Stomach poorly expressed all three subtypes relative to the other tissues. Spleen showed weak expression of both PPAR*α* and *β*, while expression of PPAR*β* and *γ* was weak in the heart. 

**Figure 10:**
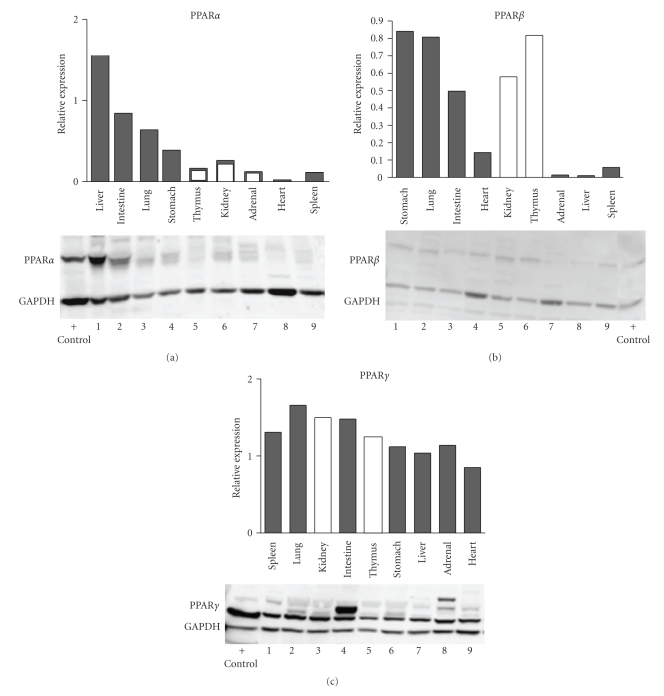
Western blots are shown in which all 9 tissues are present on each blot. On the PPAR*α* blot, all tissues shown by dark bars were from a single 91-day-old fetus, and adrenal and kidney (white bars) were from different 91-day-old fetuses, and thymus was from a 101-day-old fetus. The blots for PPAR*β* and PPAR*γ* used samples from a 91-day-old fetus (dark bars, the same set of samples for both PPAR*β* and *γ*) with kidney and thymus samples (white bars) from different fetuses (91 and 108 days, resp.). Blot images are labeled to show the location of the PPAR band, the GAPDH band, and lane containing the positive control (Hep G2 whole cell extract, Jurkat cell nuclear extract, and U937 whole cell extract, for expression of PPAR*α*, *β*, or *γ*, resp.). The densitometry data (PPAR expression normalized to GAPDH) for each gel is shown above the blot image. Lanes 1–9 contain the samples listed on the *x*-axis of the bar graphs.

Comparison of the relative levels of protein expression for each PPAR subtype is not presented. The expression of GAPDH did not change with age, making it a suitable loading control for normalization of the PPAR densitometry values from the Western blots for each tissue across age (data not shown); however, GAPDH expression was substantially different between the tissues and that makes it inappropriate to compare or rank the normalized expression between the different tissues. Even considering that the levels of GAPDH were not uniformly expressed in the different tissues, it is clear that there were different levels of PPAR*α*, *β*, and *γ* proteins in the various tissues. This can be seen in Figure 10 which illustrates the expression of PPAR proteins in all 9 tissues on single blots for each subtype. In these assays, all 9 tissues were present on each blot and, to the extent possible, the tissues on each blot were from the same 91-day old fetus. Thymus was only available from 101-, 108-, and 110-day old fetuses. Three of the cross-tissue assays (each using tissues from different fetuses) were run for each PPAR subtype and examples of the multiple tissue blots are shown in Figure 10.

## 4. Discussion

This study provides new information regarding the expression of PPAR subtypes during human fetal development. PPAR*α*, *β* and *γ* are expressed in the human fetus from embryonic days 54 to 125. Protein and mRNA for all three PPAR subtypes were detected in the 9 tissues examined in this study. In some organs, the expression of mRNA or protein changed during the developmental period examined. Relative levels of mRNA expression of the PPAR subtypes varied by tissue. In some organs, the level of mRNA expressed was comparable to or higher than that of the adult tissue. 

Human fetal expression of PPAR subtypes can be considered similar to the expression patterns reported for the laboratory rodent, reviewed in [26]. In mouse and rat, PPAR mRNA and/or protein was detected during prenatal and postnatal development for liver, kidney, heart, lung, adrenal, spleen, vertebra, tissues of the central nervous system (CNS), brain, adipose, fat, muscle, and skin. The patterns of expression varied by tissue and were dependent on developmental stage. It is difficult to make specific comparisons between developmental patterns of PPAR expression in the laboratory animal and the human fetal tissues of this study as comparisons between comparable developmental stages become complicated following the end of organogenesis [26]. In the present study, the period of human fetal development ranged from about 8 to 18 weeks, a period following organogenesis and encompassing the fetal stage of rapid growth, differentiation, and functional maturation of the organ systems. The end of organogenesis and beginning of the fetal period are generally considered to occur at the end of the eighth week of gestation [37] and a landmark of the entry to the fetal stage is the fusion of the secondary palate. Palatal fusion in human fetuses begins around embryonic day 54 and is generally complete in the 56-57-day-old fetus [37, 38]. In the mouse and rat, palatal fusion occurs on ED14-15 and 16-17, respectively, although this can vary by a day or two depending on the strain. Thus, it may be reasonable to consider the ED14 mouse, ED16 rat, and the ED54–56 human fetal tissues to be at comparable developmental stages for purposes of comparison of PPAR expression. Restricting the discussion to that specific developmental period (end of organogenesis marked by palatal fusion), the comparisons of human and rodent PPAR expression are somewhat limited. Overall, as discussed below, there are similarities, and also some differences, in the expression of PPAR in rodent and human fetuses at the end of organogenesis. 

In the ED15.5 rat liver, moderate levels of mRNA for PPAR*α* and *β* were found and PPAR*β* protein was reported in ED15 mouse liver and PPAR*γ*2 protein was detected at a slightly earlier stage (ED13) in mouse liver [39, 40]. In the present study, PPAR*α* was highly expressed in the human fetal liver and relatively abundant compared to other tissues (only intestine was higher). When evaluated across all ages, PPAR*α* and *γ* were more abundant than *β* in liver. 

Rat heart and lung expressed PPAR*α* and *β*, and PPAR*β* protein expression is reported for mouse heart and lung [40, 41]. In the present study, human fetal lung and heart had high expression of PPAR*α* and lung strongly expressed PPAR*α* and *β* relative to the other organs. In human fetal heart and lung, PPAR*α* was more abundant than *β* or *γ*, and in heart *γ* was the subtype with the least expression. 

The ED15.5 rat and 14.5 mouse kidneys expressed PPAR*α* mRNA. PPAR*β* mRNA was found in rat and PPAR*β* and *γ* were weakly detected in the mouse kidney [41, 42]. In human fetal kidney, PPAR*γ* was expressed at higher levels than *α* or *β*, and *β* was the least abundant subtype in kidney. 

We are not aware of any published data regarding expression of PPAR in thymus or spleen of the developing rodent. In the human fetal spleen, PPAR*α* and *β* were expressed at low but equivalent levels and PPAR*γ* was the most abundant subtype with relatively high expression (only those of thymus and intestine were higher). In human fetal thymus, PPAR*γ* mRNA was very abundant (higher than in any other tissue) and PPAR*β* and *α* were detected at lower levels than PPAR*γ*.

In rat GI tract, mRNAs for PPAR*α* and *β*, but not *γ* (reported as not detected), were expressed, and PPAR*β* protein was reported in mouse GI tissue [40, 41]. The present study found high expression in intestine for all PPAR subtypes relative to the other organs, and PPAR*α*, *β*, and *γ* mRNAs were at equivalent levels. Stomach, which was examined separately, had lower expression of all subtypes relative to intestine, and PPAR*γ* was the most highly expressed subtype in stomach. Huin et al. [30] examined PPAR protein expression in the fetal human digestive tract (aged 7 to 22 weeks) using immunohistochemistry and found spatial and temporal patterns of expression in esophagus, stomach, jejunum, ilium, and colon. In the present study, using qPCR and Western blotting methods, no change with age was detected in stomach for expression of PPAR*α*, *β*, or *γ*. Huin's report found slightly less PPAR*α* at 19 weeks compared to 12 and 15 weeks of age, while PPAR*β* and *γ* were slightly higher at 15 and 16 weeks, respectively, than at 12 or 19 weeks of gestation. The 19-week observations of Huin's study are just outside the age range of the present study, but the slight changes in protein reported by Huin differ from our observations of mRNA and protein at the earlier ages. The intestinal expression of PPAR reported for the various regions observed in Huin's study is similar as an overall pattern to that found in the present study; however, in the present study it was not possible to separate regions of the intestinal tract. Huin reported increasing PPAR*α* in the ileum from 12 to 22 weeks of age, similar to the increase with age in PPAR*α* protein observed from 8 to 18 weeks in the present study. Similarly, Huin reported that PPAR*β* and *γ* protein expression in the jejunum and ilium was similar across time (7–16 and 12–22 weeks, resp.), and the present study also found no significant change in protein expression of PPAR*β* or *γ* with age. 

An important finding of the present study is that fetal tissues can express PPAR at levels equivalent to those of the adult tissues (or higher in the case of PPAR*γ* in liver and PPAR*β* in thymus). However, some caution is needed as the adult data for each tissue (with the exception of liver) is based on a pooled total RNA sample from 3 donors and it is not known whether a similar outcome would be derived from a larger number of adult donors. However, in the case of liver, the data from 23 individuals supported the data from the pooled sample, that is, expressions of PPAR*α* and *β* were not significantly different in adult and fetal livers. However, the pooled sample did not detect the increased expression of PPAR*γ* in fetal liver relative to the adult, as observed in the 23 individual liver samples. Thus, the adult versus fetal comparison provides data that were previously not available and represent the only information for this endpoint. However, it is important to recognize that comparisons of these data with additional analyses from larger adult tissue sets would be desirable. 

In summary, this study is unique in providing substantial information on the expression of PPAR*α*, *β*, and *γ* during human fetal development. Among the strengths of the study are the acquisition of both protein and mRNA data from the same samples, the inclusion of multiple tissues from most fetuses, and the large number of individuals represented in the sample set. Representation of tissues across a range of ages supported an evaluation of whether PPAR expression changed as development progressed. The qPCR approach supported estimation of the relative expression of subtypes within a tissue as well as supporting comparisons of expression of each subtype across the different tissues. As mentioned in the introduction, an important aspect of this study was to provide information for use in assessing the potential for the human fetus to respond to PPAR agonists. Studies in human fetal tissues of responses to PPAR agonists are generally not feasible; thus, it is important to at least have information on the developmental expression of PPAR and how that compares to adult expression. This study contributes to our knowledge regarding the expression of PPAR during development and compares fetal and adult PPAR expression. An important finding of the study is that fetal tissues can have expression levels equivalent to those of the adult tissues (or higher in the case of PPAR*γ* in liver and PPAR*β* in thymus). The role of PPAR subtypes in the developing fetus remains unclear, but it is likely that these nuclear receptors have roles similar to those described for adult tissues, including regulation of energy homeostasis as well as lipid and glucose utilization. During the fetal stages examined in this study, the organs undergo rapid growth, differentiation, and acquisition of functionality. Exogenous agents that alter PPAR signaling in the adult, such as environmental agents, chemicals, or drugs, are capable of affecting lipid and glucose utilization, cholesterol biosynthesis, and other metabolic pathways, and these attributes make PPAR signaling an attractive target for pharmaceuticals directed at management of disease states (diabetes, metabolic syndrome, hyperlipidemia) [4, 40]. This study showed that PPAR subtypes are expressed during human fetal development in many organs and it is likely that PPAR expression and function during development are tightly regulated. It is not clear whether specific agents perturb PPAR expression or function in the fetus, whether such perturbations will have consequences or whether effects might emerge at or persist through much later life stages. However, the demonstration of expression of PPAR*α*, *β*, and *γ* in nine major organs during human fetal development renders consideration of such issues highly relevant.

## Figures and Tables

**Table 1:** Relative RNA^1^ expression for each subtype (listed from highest to lowest mean expression), characteristics^2^ of RNA and protein expression across age, and fetal mRNA expression relative to adult.

RNA abundance: high to low	RNA change with age	Fetal versus adult mRNA	Protein change with age
PPAR*α*			

Intestine	NS	NS	Increase
Liver	Increase	NS	NS
Lung	NS	NS	Decrease
Heart	NS	Lower	NS
Kidney	NS	Lower	Decrease
Adrenal	NS	NS	Decrease
Thymus	NS	NS	NS
Stomach	NS	Lower	NS
Spleen	NS	Lower	NS
PPAR*β*			

Intestine	Decrease	NS	NS
Adrenal	NS	NS	Decrease
Lung	NS	NS	NS
Kidney	NS	Lower	NS
Thymus	NS	Higher	NS
Liver	Increase	NS	NS
Heart	Decrease	Lower	NS
Spleen	NS	Lower	NS
Stomach	NS	Lower	NS
PPAR*γ*			

Thymus	NS	NS	NS
Intestine	NS	NS	NS
Spleen	NS	NS	NS
Liver	NS	Higher	NS
Kidney	NS	NS	Decrease
Lung	NS	Lower	NS
Stomach	NS	Lower	NS
Heart	NS	Lower	NS
Adrenal	Decrease	Lower	NS

^1^Relative RNA expression based on mean Ct for all samples across all ages for each tissue, listed from highest to lowest mean expression for each subtype. ^2^Change in RNA and protein expression with age shown as increased, decreased, or not significant (NS) and fetal mRNA expression compared to adult expression shown as higher, lower, or not significantly (NS) different from adult.

**Table 2:** Relative RNA expression of PPAR isotypes within each tissue.

Intestine	*α* = *β* = *γ*
Liver	*α* = *γ* > *β*
Lung	*α* > *β* = *γ*
Heart	*α* > *β* > *γ*
Adrenal	*α* = *β* > *γ*

Thymus	*γ* > *β* > *α*
Spleen	*γ* > *α* = *β*
Kidney	*γ* > *α* > *β*
Stomach	*γ* > *α* = *β*

Relative expression based on mean Ct across ages for all samples of each tissue.
