# Erratum to “Peroxisome Proliferator-Activated Receptors Alpha, Beta, and Gamma mRNA and Protein Expression in Human Fetal Tissues”

**DOI:** 10.1155/2010/627284

**Published:** 2011-05-26

**Authors:** Barbara D. Abbott, Carmen R. Wood, Andrew M. Watkins, Kaberi P. Das, Christopher S. Lau

**Affiliations:** Toxicity Assessment Division, Developmental Toxicology Branch, National Health and Environmental Effects Research Laboratory, (MD-67) Office of Research and Development, US Environmental Protection Agency, Research Triangle Park, NC 27711, USA

In the above-mentioned paper, the expression of PPAR*γ* was incorrectly determined based on the quantification of a 50 kD band on the Western blots. This band aligned with what was believed to be a positive control band in the U937 whole cell extract, a control reagent recommended by the supplier of the primary antibody, SC-7196 (Santa Cruz Biotechnologies, Santa Cruz, CA). Based on recent information described by Foreman et al. [1], it is clear that this band is not PPAR*γ* but was a nonspecific immunoreactive protein detected by SC-7196. This nonspecific protein was abundantly detected by SC-7196 in U937 and COS-1 cells as well as across all human fetal protein samples. Immunoprecipitation of COS-1 cell lysate using agarose-conjugated SC-7196 resulted in a single band on a Coomassie Blue-stained gel. This band was subjected to digestion, peptide extraction, and sequence analysis using MALDI-MSMS, and the protein was identified as cytoplasmic actin with a decisive score (human SwissProt database, 60% protein coverage using the 15 highest scoring peptide groups and two lower scoring but acceptable peptides). 

A specific band for PPAR*γ*, (calculated molecular weight for human PPAR*γ*1 = 54.55 kD) was identified on our Western blots by performing new experiments in which in vitro translated human PPAR*γ*1 (provided by J. Peters, Pennsylvania State University) was compared with human fetal tissue lysates (Figure 1). These experiments also included COS-1 cell lysate as a negative control and U937 cell lysate. The Western blots of the fetal tissues were all reanalyzed using the ~55 kD band that aligned with the in vitro translated human PPAR*γ*1. Based on this reanalysis, the expression of PPAR*γ* protein shown in (c) of Figures  1–9 are replaced by Figure 2. The data summary described in Table  1 of the above-mentioned paper regarding the change in PPAR*γ* protein expression with fetal age is replaced by Table 1. 

The authors regret this unexpected error. The clarification of the recognition patterns of this primary antibody should be of value to investigators interested in detecting PPAR*γ* protein.

## Figures and Tables

**Figure 1:**
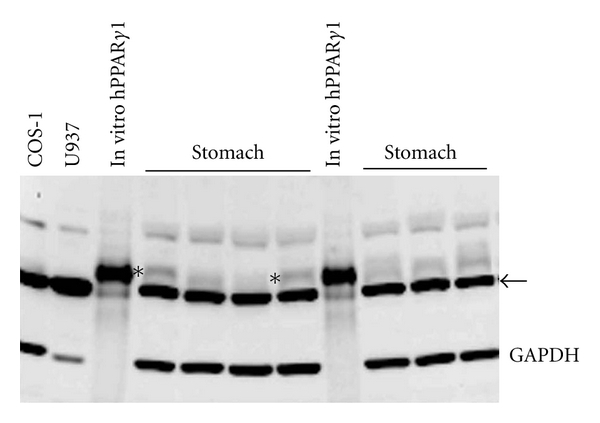
Western blot showing the comparison of banding patterns in COS-1 cell lysate, U937 cell lysate, in vitro translated human PPAR*γ*1, and tissue lysate from human fetal stomach samples. The ~55 kD band of human PPAR*γ*1 and corresponding band in stomach tissue lysate is marked with an asterisk (∗). The nonspecific, cytoplasmic actin band is marked with an arrow.

**Figure 2:**
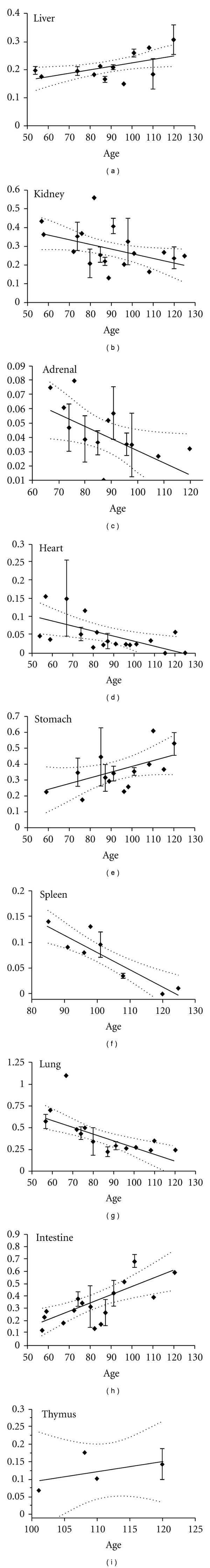
PPAR*γ* protein expression is shown across the fetal age range for each tissue. Western blot density normalized to glyceraldehyde-3-phophate dehydrogenase (GAPDH). If only one sample was available for a particular age, then an error term could not be calculated and no SEM bar is shown. Regression analysis evaluated change with age. Dashed lines are the 95% confidence interval.

**Table 1:** 

Tissue	Protein change with age	
Thymus	NS	
Intestine	Increase	*P* < .01
Spleen	Decrease	*P* < .001
Liver	Increase	*P* < .05
Kidney	Decrease	*P* < .05
Lung	Decrease	*P* < .001
Stomach	NS	
Heart	Decrease	*P* < .05
Adrenal	Decrease	*P* < .05

NS = no significant change with age.

## References

[1] Foreman JE, Sorg JM, McGinnis KS Erratum: to Regulation of peroxisome proliferator-activated receptor-*β*/*δ* by the APC/*β*-CATENIN pathway and nonsteroidal antiinflammatory drugs.

